# Breaking barriers: predictors of condom use negotiation among adolescent girls and young women in Mozambique

**DOI:** 10.1186/s12889-025-22315-0

**Published:** 2025-03-18

**Authors:** Ololade Julius Baruwa

**Affiliations:** Centre for Social Sciences Research, University of Cape Town, Cape Town, South Africa

**Keywords:** Condom use, Negotiation, DHS, Theory of planned behaviour, Inequitable gender attitudes, HIV testing

## Abstract

**Background:**

Effective condom use negotiation is crucial for reducing HIV risk and unintended pregnancies. While studies have emphasized condom use, they often overlook the negotiation process, a critical factor in mitigating sexual health risks. This study examines factors influencing condom use negotiation among adolescent girls and young women (AGYW) in Mozambique.

**Methods:**

Data from the 2022–23 Mozambique Demographic and Health Survey (MZDHS) were analyzed, focusing on 2,624 AGYW aged 15–24. Condom use negotiation, defined as the ability to ask a partner to use a condom, was treated as a binary variable. Ten independent variables measured as binary, nominal, or ordinal were included: age, education, residence, wealth status, religion, early sexual debut, adolescent motherhood, multiple sexual partners, HIV testing, and inequitable gender attitudes. Multivariable logistic regression identified the predictors of condom use negotiation, with interaction terms assessing whether gender attitudes and condom negotiation varied by age. Average marginal effects were calculated to evaluate the impact of these factors.

**Results:**

Only 35.11% of AGYW reported the ability to negotiate condom use. Multivariate analysis showed that AGYW with secondary or higher education (adjusted Odds ratio [aOR] = 2.17, 95% Confidence Intervals [CI] = 1.48–3.17; *p* < 0.001) and those from rich households wealth index (aOR = 2.51, 95% CI = 1.81–3.48; *p* < 0.001) were more likely to negotiate. HIV testing was also associated with higher odds of condom use negotiation (aOR = 1.39, 95% CI = 1.01–1.91; *p* = 0.043). Conversely, inequitable gender attitudes reduced the likelihood of condom use negotiation (aOR = 0.62, 95% CI = 0.46–0.83; *p* = 0.001). AGYW aged 15–19 with inequitable attitudes had the lowest predicted probability of condom use negotiation (31%).

**Conclusion:**

Findings emphasize the role of education, wealth, gender attitudes, and HIV testing in enhancing condom use negotiation among AGYW in Mozambique. Public health initiatives should address these factors to strengthen negotiation skills and support HIV prevention efforts.

## Introduction

Negotiating condom use is a critical aspect of safe sexual practices, particularly in Mozambique, where the prevalence of Human Immunodeficiency Virus (HIV) and unintended pregnancies remains a significant public health concern [1, 2]. Condom use negotiation refers to the process by which individuals in a sexual relationship communicate and decide on the use of condoms during sexual activity [3, 4]. This negotiation involves both verbal and non-verbal communication and is considered a set of behaviours rather than a singular act [4]. Effective condom use negotiation is crucial for preventing HIV, other sexually transmitted infections (STIs), and unintended pregnancies [5, 6].

Mozambique has one of the highest HIV prevalence rates globally, with 12.4% of individuals aged 15–49 living with HIV, and young women aged 15–24 are disproportionately affected [1]. Additionally, approximately 88% of all pregnancies among females aged 15–49 years in Mozambique are unintended, leading to adverse health, economic, and social outcomes [2]. Failure to consistently use condoms has been identified as a major factor contributing to the heightened risk of HIV and other sexually transmitted infections (STIs) [7, 8]. Unprotected heterosexual intercourse remains the predominant mode of HIV transmission and a key driver of unintended pregnancies [9]. Misperceptions of low HIV risk, concerns about reduced sexual pleasure, economic constraints, and stigma or embarrassment associated with condom use are significant deterrents [10, 11]. These challenges are further exacerbated by inequitable gender attitudes and limited autonomy among adolescent girls and young women (AGYW), particularly in sexual decision-making processes.

The Theory of Planned Behaviour (TPB) offers a robust framework for understanding the behavioural dynamics influencing condom use negotiation. According to TPB, *attitudes*,* subjective norms*,* and perceived behavioural control* collectively shape an individual’s ability to negotiate condom use. Positive *attitudes*, such as recognizing condoms’ effectiveness in preventing HIV and unintended pregnancies, improve negotiation likelihood. Conversely, negative *attitudes*, including concerns about reduced intimacy and sexual pleasure, present significant barriers [12, 13]. *Subjective norms*, including partner characteristics, community expectations, and cultural norms, significantly influence AGYW’s negotiation power. AGYW in relationships with older or economically dominant partners face heightened challenges in asserting their preferences. Supportive norms, such as peer encouragement or public health campaigns promoting safe sex, have shown promise in empowering AGYW to negotiate [14]. *Perceived behavioral control*, or confidence in overcoming barriers, is bolstered by education and prior negotiation successes but undermined by fear of conflict, violence, or cultural expectations [14].

Sociodemographic and behavioural factors, such as age, education, socio-economic status, risky sexual behaviours, and gender attitudes, are critical in understanding condom use negotiation [14–17]. Younger AGYW often face challenges like limited autonomy, lower sexual health knowledge, and power imbalances in relationships, whereas older AGYW, particularly those with higher education or economic independence, tend to have more negotiation power [18]. Inequitable gender norms, which reinforce male dominance, may further hinder AGYW’s ability to assert preferences, with younger AGYW doubly disadvantaged when exposed to such norms [19]. The interaction between age and gender attitudes is crucial, as it shapes the negotiation process and should be considered in targeted interventions.

While research has explored inconsistent condom use among AGYW [10, 13] and broader contraceptive decision-making [17, 20], limited studies focus on condom use negotiation among ever-partnered AGYW. Understanding the factors influencing condom use negotiation is crucial, as AGYW face unique interpersonal and cultural barriers that may limit their ability to advocate for safer sex practices. This study addresses this gap by investigating the predictors of condom use negotiation among AGYW in Mozambique and exploring the interaction between age and gender attitudes. The findings will provide valuable insights to inform policies and interventions aimed at improving sexual and reproductive health outcomes for AGYW in Mozambique.

## Data and methods

This study utilized data from the 2022–23 Mozambique Demographic and Health Survey (MZDHS), a cross-sectional survey conducted by the National Institute of Statistics (INE) in collaboration with the Ministry of Health (MISAU) and the National Health Institute (INS), with technical assistance from the ICF Macro MEASURE DHS program. The sampling frame was based on the 2017 General Population and Housing Census (GPH), where manageable enumeration areas (EAs) were designated as Primary Sampling Units (PSUs). The 2022–23 MZDHS aimed to provide national, urban, rural, and provincial-level estimates, covering Mozambique’s ten provinces (Niassa, Cabo Delgado, Nampula, Zambézia, Tete, Manica, Sofala, Inhambane, Gaza, and Maputo) as well as the capital, Maputo City, which has provincial status. A two-stage stratified sampling method was used. In the first stage, 619 enumeration areas (EAs) were selected with probability proportional to size based on the number of households in each stratum. This included 232 urban EAs and 387 rural EAs. In the second stage, 26 households were systematically selected within each EA [21].

Five types of questionnaires were administered: household, individual women’s, individual men’s, caregiver’s, and biomarker questionnaires. This study used data from the individual women’s questionnaire, which collected information on background characteristics (e.g., age, education, and residence), birth history, child mortality, family planning, marriage, sexual activity, fertility preferences, and HIV/AIDS knowledge [21]. Data was collected nationwide between July 27, 2022, and February 2023. Of the 13,183 women successfully interviewed, 2,624 were aged 15–24 years, sexually active and responded to the question on condom use negotiation, forming the sample for this study. A quick tabulation of the data also revealed that only married or cohabiting women answered the condom use negotiation question.

### Outcome variable

The outcome variable in this study is condom use negotiation, which measures whether the respondent can ask their partners to use a condom. Condom use negotiation was coded as 1 if the respondent indicated “yes” and coded as 0 if the respondent indicated “no” or responded with “don’t know/not sure/depends.”

### Independent variables

The selection of independent variables for this study was guided by existing research [15–17, 20]. Ten variables, identified for their significance in influencing inconsistent condom use, non-use, and condom use negotiation, were included based on their relevance and availability in the dataset. These variables are *age*,* education*,* place of residence*,* wealth status*,* religion*,* early sexual debut*,* adolescent motherhood*,* multiple sexual partners*,* HIV testing*,* and inequitable gender attitudes.*

*Age* was defined as the current age of AGYW and categorized into “15–19” and “20–24.” *Education* was defined as the highest level of education completed by the participants and was classified into three categories: “no education,” “primary,” and “secondary or higher.” *Place of residence* was categorized as either “urban” or “rural,” while the *wealth index* was measured in three groups: “poor,” “middle,” and “rich.” Religion was categorized into four groups: “Catholic,” “Muslim,” “Other Christians,” and “No religion.” The “Other Christians” category includes Zion, Evangelical/Pentecostal, and Anglican denominations. Sixteen AGYW who reported practicing other religions were excluded from the analysis due to the small sample size.

Behavioural and gender attitude variables were also considered in the analysis. *Early sexual debut* was defined as initiating sexual intercourse before the age of 16 and measured as “no” or “yes.” The decision to use 16 years as the threshold was informed by international guidelines, such as those from the World Health Organization (WHO) and a prior study [22, 23]. *Adolescent motherhood* was defined as the conception of the first child before the age of 20. *Multiple sexual partnerships* referred to having more than one sexual partner in a lifetime and were measured as “no” or “yes.” *HIV testing* was defined as having ever been tested for HIV and measured similarly. *Inequitable gender attitudes* were assessed based on agreement with at least one justification for a husband beating his wife. Participants were considered to have inequitable gender attitudes if they agreed with any of the following justifications: (i) if the wife goes out without informing her husband, (ii) if she neglects the children, (iii) if she argues with him, (iv) if she refuses to have sex with him, or (v) if she burns the food.

### Data analyses

Five analytical steps were undertaken to examine the predictors of condom use negotiation. First, descriptive statistics were computed for the outcome variable (condom use negotiation) and the independent variables, including age, education, place of residence, wealth status, religion, early sexual debut, adolescent motherhood, multiple sexual partners, HIV testing, and inequitable gender attitudes. Second, a univariable logistic regression model was employed. Third, all variables found to be significant in the univariable analysis were included in the multivariable logistic regression model to identify the factors associated with condom use negotiation. Fourth, an interaction term between age and inequitable gender attitudes was tested to determine whether the association between gender attitudes and condom use negotiation varied by age. Finally, average marginal effects (AMEs) were used to compute adjusted predicted probabilities of condom use negotiation across all combinations of age and gender attitudes.

Weighted estimates were derived to accommodate the DHS’s complex design. All data management and statistical analyses were conducted using Stata 16. The variables for inequitable gender attitudes and religion had 29 and 16 missing data points, respectively, but this missing data did not affect the analysis outcomes. Listwise deletion was used to handle the missing data. Results were interpreted using odds ratios, with statistical significance set at a *P*-value < 0.05 and a confidence interval of 95%.

## Results

### Characteristics of the study population

We analyzed data from 2,624 AGYW aged 15–24 in Mozambique (see Table 1). Among the participants, 65.71% were aged 20–24, and 23.28% had no formal education. The majority (73.63%) of AGYW lived in rural areas, 49.51% belonged to the poor household wealth index, and 31.71% identified as practising the Catholic religion.

Condom use negotiation was reported by only 35.11% of AGYW aged 15–24. Early sexual debut was reported by 57.28% of participants. Adolescent motherhood was reported by 74.67% of the AGYW. Additionally, 46.69% of AGYW reported having had more than one sexual partner in their lifetime, including their spouse. Regarding HIV testing, 66.73% of AGYW reported having ever been tested for HIV. Finally, 23.45% of the participants reported holding inequitable gender attitudes, agreeing with at least one justification for a husband beating his wife.

**Table 1:** Characteristics of the study population

	Proportion (Weighted %)	95% CI	Sample size (*n*)
**Socio-demographic characteristics**			
**Age**			
15–19 20–24	34.29 65.71	31.99–36.67 63.33–68.01	2624
**Education**			
No education Primary Secondary or higher	23.28 53.83 22.89	20.80-25.95 50.79–56.84 20.57–25.39	2624
**Place of residence**			
Urban Rural	26.37 73.63	23.47–29.48 70.52–76.53	2624
**Wealth status**			
Poor Middle Rich	49.51 20.08 30.41	45.69–53.34 17.68–22.72 27.29–33.72	2624
**Religion**			
Catholic Muslims Other Christians No religion	31.71 24.18 36.34 7.77	28.37–35.24 20.99–27.69 33.47–39.30 5.91–10.16	2608
**Gender attitude and behavioural characteristics**			
Condom use negotiation	35.11	32.52–37.79	2624
Early sexual debut	57.28	54.55–59.96	2624
Adolescent motherhood	74.67	72.45–76.77	2624
Multiple sexual partners	46.69	44.04–49.37	2624
HIV testing	66.73	63.90-69.44	2624
Inequitable gender attitude	23.45	20.72–26.42	2595

### Univariable logistic regression of factors associated with condom use negotiation

In the univariable logistic regression analysis, all independent variables were associated with condom use negotiation (see Table 2). Specifically, AGYW aged 20–24 years were more likely to report negotiating condom use (aOR = 1.35, 95% CI = 1.09–1.68; *p* = 0.006). AGYW with primary education (aOR = 1.52, 95% CI = 1.13–2.07; *p* = 0.006) and secondary education (aOR = 4.88, 95% CI = 3.54–6.72; *p* < 0.001) were also more likely to negotiate condom use. Similarly, AGYW from households with a middle wealth index (aOR = 2.28, 95% CI = 1.64–3.18; *p* < 0.001) and a rich wealth index (aOR = 4.30, 95% CI = 3.26–5.68; *p* < 0.001) were more likely to negotiate condom use. In contrast, AGYW from rural areas were less likely to negotiate condom use (aOR = 0.39, 95% CI = 0.31–0.50; *p* < 0.001). Regarding religion, AGYW who identified as other Christians were more likely to negotiate condom use (aOR = 1.41, 95% CI = 1.07–1.84; *p* = 0.014).

Regarding early sexual debut, AGYW who initiated sexual intercourse before age 16 were less likely to negotiate condom use (aOR = 0.80, 95% CI = 0.64–0.99; *p* = 0.043). AGYW who became mothers before age 20 were also less likely to negotiate condom use (aOR = 0.77, 95% CI = 0.60–0.97; *p* = 0.027). Concerning multiple sexual partnerships, AGYW reporting lifetime multiple sexual partnerships were more likely to negotiate condom use (aOR = 1.23, 95% CI = 1.01–1.50; *p* = 0.036). AGYW who had undergone HIV testing were more likely to negotiate condom use (aOR = 2.31, 95% CI = 1.76–3.03; *p* < 0.001). Finally, AGYW with inequitable gender attitudes were less likely to negotiate condom use (aOR = 0.52, 95% CI = 0.39–0.69; *p* < 0.001).

### Multivariable logistic regression of factors associated with condom use negotiation

In the multivariable logistic regression analysis, four factors were associated with condom use negotiation (see Table 2). AGYW with secondary or higher education were more likely to negotiate condom use (aOR = 2.17, 95% CI = 1.48–3.17; *p* < 0.001). AGYW from households with a middle wealth index (aOR = 1.96, 95% CI = 1.42–2.71; *p* < 0.001) and a high wealth index (aOR = 2.51, 95% CI = 1.81–3.48; *p* < 0.001) were more likely to engage in condom use negotiation. Additionally, AGYW who had undergone HIV testing were more likely to negotiate condom use (aOR = 1.39, 95% CI = 1.01–1.91; *p* = 0.043). On the other hand, AGYW who held inequitable gender attitudes were less likely to negotiate condom use (aOR = 0.62, 95% CI = 0.46–0.83; *p* = 0.001).

**Table 2:** Multivariable logistic regression analysis of factors associated with condom use negotiation

	uOR (95% CI)	*P*-Value	aOR (95% CI)	*P*-Value
**Age**				
15–19 20–24	1 1.35 (1.09–1.68)	0.006	1 1.25 (0.97–1.60)	0.086
**Education**				
No education Primary Secondary or higher	1 1.52 (1.13–2.07) 4.88 (3.54–6.72)	0.006 < 0.001	1 1.19 (0.84–1.69) 2.17 (1.48–3.17)	0.320 < 0.001
**Place of residence**				
Urban Rural	1 0.39 (0.31–0.50)	< 0.001	1 0.92 (0.70–1.21)	0.538
**Wealth status**				
Poor Middle Rich	1 2.28 (1.64–3.18) 4.30 (3.26–5.68)	< 0.001 < 0.001	1 1.96 (1.42–2.71) 2.51 (1.81–3.48)	< 0.001 < 0.001
**Religion**				
Catholic Muslims Other Christians No religion	1 0.82 (0.58–1.14) 1.51 (1.15–1.98) 1.00 (0.61–1.64)	0.233 0.003 0.985	1 0.70 (0.49–1.01) 1.08 (0.80–1.46) 0.98 (0.63–1.52)	0.058 0.628 0.923
**Early sexual debut**				
No Yes	1 0.80 (0.64–0.99)	0.043	1 1.08 (0.84–1.38)	0.542
**Adolescent motherhood**				
No Yes	1 0.77 (0.60–0.97)	0.027	1 0.80 (0.61–1.06)	0.119
**Multiple sexual partners**				
No Yes	1 1.23 (1.01–1.50)	0.036	1 0.98 (0.77–1.24)	0.846
**HIV testing**				
No Yes	1 2.31 (1.76–3.03)	< 0.001	1 1.39 (1.01–1.91)	0.043
**Inequitable gender attitude**				
No Yes	1 0.52 (0.39–0.69)	< 0.001	1 0.62 (0.46–0.83)	0.001

### Interaction effect of age and inequitable gender attitudes on condom use negotiation

Table 3 presents the analysis of the interaction effect between age and inequitable gender attitudes on condom use negotiation among adolescent girls and young women (AGYW) aged 15–24. The results indicate that age does not significantly moderate the relationship between inequitable gender attitudes and condom use negotiation.

Table 4 displays the average marginal effects from the moderation analysis of condom use negotiation, expressed as predicted probabilities. Overall, the predicted probability of condom use negotiation was below 50% across all four combinations of age and gender attitudes. The lowest predicted probability (31%) was observed among AGYW aged 15–19 with inequitable gender attitudes, followed by 33% among AGYW aged 20–24 with inequitable gender attitudes. The highest predicted probability (46%) was observed among AGYW aged 20–24 with equitable gender attitudes.

**Table 3:** Logistic regression model for condom use negotiation with interaction effects for age and inequitable gender attitudes

	aOR (95% CI)	*P*-Value
**Age**		
15–19 20–24	1 1.30 (0.99–1.70)	0.057
**Inequitable gender attitude**		
No Yes	1 0.71 (0.43–1.16)	0.173
**Age##inequitable gender attitudes**		
15–19, equitable gender attitudes 20–24, inequitable gender attitudes	1 0.82 (0.46–1.47)	0.503
**Education**		
No education Primary Secondary or higher	1 1.19 (0.84–1.69) 2.16 (1.48–3.16)	0.320 < 0.001
**Place of residence**		
Urban Rural	1 0.92 (0.70–1.21)	0.538
**Wealth status**		
Poor Middle Rich	1 1.97 (1.43–2.71) 2.01 (1.80–3.47)	< 0.001 < 0.001
**Religion**		
Catholic Muslims Other Christians No religion	1 0.70 (0.49–1.01) 1.08 (0.80–1.46) 0.98 (0.63–1.52)	0.058 0.624 0.920
**Early sexual debut**		
No Yes	1 1.08 (0.84–1.38)	0.540
**Adolescent motherhood**		
No Yes	1 0.80 (0.61–1.05)	0.109
**Multiple sexual partners**		
No Yes	1 0.98 (0.77–1.25)	0.870
**HIV testing**		
No Yes	1 1.39 (1.01–1.91)	0.042

The symbol “##” denotes interaction terms between age and inequitable gender attitudes

**Table 4:** Percentage predicted probabilities of condom use negotiation among AGYW by age and inequitable gender attitudes

	Probability (%)	95% CI	*P*-Value
15–19, equitable gender attitudes 15–19, inequitable gender attitudes 20–24, equitable gender attitudes 20–24, inequitable gender attitudes	0.39 0.31 0.46 0.33	0.34–0.44 0.21–0.42 0.40–0.51 0.26–0.40	< 0.001 < 0.001 < 0.001 < 0.001

## Discussion

Efforts to develop effective interventions to reduce HIV/AIDS and unwanted pregnancies among AGYW in Mozambique require an understanding of important factors that interventions should target or strengthen. The present study sought to contribute to the existing body of knowledge regarding the predictors of condom-use negotiation among AGYW using the 2022-23 Mozambique Demographic and Health Survey data. This study highlights critical factors associated with condom use negotiation among AGYW in Mozambique. These factors, including education, household wealth index, HIV testing, and gender attitudes, offer insights into the determinants of sexual health agency in this population.

The finding that AGYW with secondary or higher education were more likely to negotiate condom use underscores the pivotal role of education in fostering empowerment and sexual and reproductive health rights (SRHRs). Higher education likely equips individuals with knowledge, communication skills, and confidence, which are essential for advocating safer sexual practices. This aligns with existing research, which emphasizes that education enhances HIV awareness and promotes self-efficacy in sexual health decision-making [24]. However, education alone may not fully translate into condom use negotiation in contexts where cultural and relational dynamics constrain AGYW’s agency. For instance, studies have shown that even educated women may face challenges such as partner resistance or societal norms that limit open discussion about sexual health [25, 26]. These nuances suggest that while education is foundational, complementary interventions, such as peer-led programs or community engagement efforts, are necessary to amplify its impact.

The positive association between middle and rich household wealth and condom use negotiation reflects the role of economic stability in enhancing health agency. Wealthier households provide AGYW with access to resources and health information and reduced dependence on partners, fostering autonomy in decision-making [18, 27]. Financial security enables AGYW to exercise greater control over their sexual and reproductive health, promoting self-efficacy in negotiating safer sex practices [27]. This autonomy is critical in the context of HIV prevention, where negotiation skills and the ability to refuse unsafe sex can be crucial in reducing the risk of transmission. Moreover, economic empowerment enhances women’s ability to make health-related decisions [27]. In addition, women with access to economic resources are more likely to make independent decisions regarding their health, including the use of contraception and negotiation of condom use [18]. Similarly, a study in Morocco underscores the importance of economic empowerment in enabling women to access sexual and reproductive health services despite the challenges posed by restrictive gender norms and partner resistance [25].

The association between HIV testing and condom use negotiation underscores the role of health-seeking behaviours in enhancing sexual health awareness and promoting proactive prevention strategies. Engaging in HIV testing not only facilitates early detection and treatment but also provides opportunities for counselling on risk reduction, including the consistent use of condoms. Moreover, this finding aligns with UNAIDS [28], which highlights that individuals who engage in HIV testing are more likely to receive counseling on risk reduction, including condom use. By integrating HIV testing with comprehensive sexual health education and open communication, individuals are better equipped to negotiate condom use, thereby reducing the risk of HIV transmission. Moreover, a study found that individuals who communicated with someone other than a parent or guardian about HIV/AIDS were more likely to have higher self-efficacy in condom use [29]. This suggests that open discussions about HIV can empower individuals to negotiate condom use more effectively and aligns with an existing study which reported that comprehensive HIV/AIDS knowledge is significantly associated with safer sex negotiation among AGYW in sub-Saharan Africa [30]. However, the association between HIV testing and condom use negotiation is borderline (*p* = 0.043) in this study. While statistically significant, the *p*-value suggests the evidence is not strong enough for definitive conclusions. This requires cautious interpretation and consideration of potential confounding factors. Larger studies with more rigorous methodologies are needed to clarify this relationship.

The association between inequitable gender attitudes and condom use negotiation highlights the detrimental impact of patriarchal norms on AGYW’s sexual health agency. Gender inequities limit AGYW’s ability to assert their preferences in sexual relationships, often placing them at heightened risk of HIV and other sexually transmitted infections. This finding aligns with a previous study, which identified patriarchal norms as a significant barrier to women’s autonomy [19]. The predicted probabilities further illustrate this disparity: AGYW with inequitable gender attitudes have lower probabilities of condom use negotiation, particularly among younger age groups (31% for those aged 15–19). This suggests that inequitable attitudes are deeply entrenched and may reflect limited exposure to gender-equity interventions during formative years. Addressing these attitudes requires targeted intervention programs that engage both AGYW and their male counterparts to challenge harmful norms and foster equitable relationships.

### Limitations and strengths

Several limitations should be considered when interpreting the findings. First, self-reported measures of condom negotiation and sexual behaviours are subject to social desirability bias, which may lead to inaccurate reporting; future research could address this through triangulation methods. Second, the cross-sectional design limits causal inferences, as only associations can be established. Third, certain key variables were excluded due to sample size constraints. For example, sexual violence data was omitted as the domestic violence module was administered to only one woman aged 18 or above per household. Additionally, information on cigarette or tobacco use, alcohol consumption, and partner age difference was not included due to insufficient sample size or lack of data in the Mozambique DHS. Excluding these variables may limit the comprehensiveness of the findings, as previous research has demonstrated their significant influence on sexual decision-making and negotiation dynamics [6, 14, 18]. Fourth, the data used did not specify whether the question on condom use negotiation referred to regular partners or casual partners. Lastly, there was no data on whether those who negotiated condom use followed through with using condoms. Despite these limitations, the study offers valuable insights to inform policy and program interventions. The inclusion of women aged 15–24 helps mitigate recall bias.

## Conclusion

This study highlights key factors influencing condom use negotiation among AGYW in Mozambique. Higher education levels and socioeconomic status, as indicated by a middle or high household wealth index, significantly increased the likelihood of condom use negotiation. Additionally, AGYW who had undergone HIV testing were more proactive in negotiating condom use, reflecting the potential influence of awareness and engagement with health services on sexual decision-making. Conversely, inequitable gender attitudes emerged as a critical barrier to condom use negotiation, underscoring the persistent challenge posed by gender norms that undermine female agency in sexual relationships. Notably, the interaction between age and gender attitudes revealed that younger AGYW with inequitable gender attitudes were the most disadvantaged group, with the lowest predicted probability of condom use negotiation. Even among older AGYW, equitable gender attitudes considerably improved the likelihood of negotiation, though the overall probability remained below 50%.

These findings underscore the urgent need for targeted interventions that address gender norms, promote equitable attitudes, and empower AGYW through education and socioeconomic support. Furthermore, integrating HIV testing with empowerment initiatives could enhance AGYW’s confidence and ability to navigate sexual health negotiations. Tailored strategies focusing on younger AGYW and those in disadvantaged households are crucial for improving sexual and reproductive health outcomes in this vulnerable population.

## Data Availability

The study used secondary data from dhsprogram.org, which is publicly available at https://dhsprogram.com/data/available-datasets.cfm.
